# Oral Health Status and Oromucosal Lesions in Patients Living with HIV/AIDS in India: A Comparative Study

**DOI:** 10.1155/2014/480247

**Published:** 2014-08-20

**Authors:** Sandeep Kumar, Prashant Mishra, Shilpa Warhekar, Bhuvnesh Airen, Deepika Jain, Shaijal Godha

**Affiliations:** Department of Public Health Dentistry, Sri Aurobindo College of Dentistry, India

## Abstract

Oral health status of HIV positive individuals is in poor condition which may be a sequela of variety of factors. This study was aimed at assessing and comparing the oral health status and oromucosal lesions between HIV positive and negative individuals in India. A total of 126 HIV positive and 532 HIV negative individuals were recruited for the study. Oral health status and oromucosal lesions were recorded using WHO oral health assessment form (1997). Data was analyzed using chi-square and independent sample student's *t* test. Majority (85.7%) of people suffering from HIV belonged to lower socioeconomic status. The mean for DMFT score was found to be significantly higher in HIV positive individuals (12.83 ± 9.6) as compared to HIV negative individuals (8.34 ± 7.6) (*P* value < 0.0001). Nearly 75% of HIV positive individuals showed oromucosal lesions with candidiasis (36%) being the most common. Nearly 50% of HIV positive individuals had community periodontal index (CPI) and loss of attachment (LOA) score >2. In conclusion HIV positive individuals have poor oral health status and poor periodontal status compared to control group. Effective policies need to be drafted to take care of the oral health of this high risk group.

## 1. Introduction

The worst disease that can ever happen to mankind is his/her inability to resist other diseases. HIV is a condition caused by human immunodeficiency virus. The condition gradually destroys the immune system which makes it harder for the body to fight infections [1].

Human immunodeficiency virus is a major global health problem. Based on HIV Sentinel Surveillance 2008-09, it is estimated that India has an adult prevalence of 0.31 percent with 23.9 lakh people infected with HIV, of which, 39 percent are female and 3.5 percent are children [2]. Not only does it destroy the immunity of the infected person but also it results in an elevated tendency to acquire and manifest diseases that are considered usually resistible by the normal human body. With the inclusion of depletion in health, such a state also depletes the quality of living, which results in further complications as far as oral disease states are concerned.

Oral lesions that are associated with this disease are important, since they affect the quality of life of the patient and are useful markers of disease progression and immunosuppression [3]. In developed countries, oral lesions in HIV infection have been well documented but, in developing countries like India, there are fewer reports on oral lesions that have been documented. There is limited literature available wherein the oral health status and oromucosal lesions in patients suffering from HIV/AIDS have been compared to general population. Thus, this study was carried out with the objective of assessing and comparing the oral health status and oromucosal lesions between the HIV positive and HIV negative individuals in central India. The study findings would enable policy makers to draft effective policies to improve oral health status of the vulnerable group.

## 2. Methods

This cross-sectional study was carried at Saathi Home Care Institution, Ujjain, central India. A total of 126 HIV positive individuals fulfilled the inclusion criteria and were included in the study. All individuals who were more than 15 years of age and above, present on the day of survey and willing to participate, were included in the study. Those patients who were not willing to participate were excluded from the study. A total of 532 HIV negative patients served as control group. Prior to the study a well-informed consent was taken from the participants and ethical clearance to conduct the study was taken from the Institutional Review Board, Sri Aurobindo Institute of Medical Sciences, Indore, India.

A questionnaire was prepared that collected information on sociodemographic data, oral hygiene practices, adverse habits, and years of drug use. The questionnaire was translated into local language. The back translation of questionnaire was done and verified by experts in both languages. The validity of the questionnaire was checked and verified and it was found to be good. The questionnaire was distributed by a single examiner to the participants who voluntary agreed to participate in the study. Any difficulties during the selection of choices were clarified by the same examiner and uncompleted questionnaire was omitted from the study and replaced by another patients' questionnaire. To eliminate interviewer's bias, data collection, questionnaire, and visual examination were carried out by the same examiner. Socioeconomic status was calculated according to modified Kuppuswamy scale [4]. Based upon patients education, occupation, and monthly income, the socioeconomic status of the patient was tabulated and compiled into upper, middle, and lower classes.

Dentition status, oral-mucosal condition, and periodontal status were assessed using WHO oral health proforma (1997) [5]. Caries was assessed as per World Health Organization (1997) guidelines under natural daylight by a single calibrated examiner using a mouth mirror and community periodontal index probe [5]. Buccal, lingual, occlusal, mesial, and distal surfaces of all teeth were examined for signs of caries. The number of decayed teeth, missing teeth, and filled teeth was identified based on dentition status and decayed, missing, and filled teeth (DMFT) score was obtained by adding decayed teeth (DT), missing teeth (MT), and filled teeth (FT). Oral mucosa was checked using mouth mirror and the oral conditions were categorized as per WHO oral health survey assessment form (1997) [5]. Community periodontal index was used to assess periodontal status as recommended by WHO oral health assessment form [5].

After the clinical findings were recorded, an oral health talk was given to the patients suffering from HIV for duration of fifteen minutes with the help of a model and toothbrush. The health talk primarily focused on oral hygiene maintenance.

All statistical analyses were performed using SPSS version 20 and *P* value ≤0.05 was taken as statistically significant. Sample size was calculated using 80% power of test and 0.5 type I error (*α*). Chi-square test was used to see the difference in categorical data between two groups whereas quantitative data was analyzed using independent sample *t*-test.

## 3. Results

The mean age of HIV positive individuals was 36.99 ± 9.24 years and it was significantly not different from HIV negative individuals (39.62 ± 15.47 years). Amongst the patients suffering from HIV, 60.3% were males while 39.7% were females. Amongst the HIV negative group, 56.4% were males while 43.6% were females.

Majority (85.7%) of the people suffering from HIV belonged to lower socioeconomic status. 65.9% were using toothbrush and paste for oral hygiene maintenance. Nearly 80% were under some form of drug therapy. On the contrary, nearly 50% of the HIV negative individuals belonged to middle class and were not under any form of drug therapy. The differences were found to be significantly associated between the two groups (*P* value <0.05) (Table 1).

The mean number of decayed teeth (DT), missing teeth (MT), and overall DMFT score were higher in HIV positive groups compared to HIV negative groups. The mean number of filled teeth (FT) was higher in HIV negative group compared to HIV positive. The differences were found to be significantly associated between the two groups (*P* value <0.05) (Table 2).

Significant difference was observed in the prevalence of oromucosal condition between the HIV positive and negative groups. 74.6% of the people who were suffering from HIV showed some form of oromucosal conditions. Candidiasis was the most common condition seen in 36.5% of the patients followed by ulceration (11.9%). On the contrary, nearly 66% of the HIV negative group did not show any oromucosal condition (Table 3).

There was a significant difference between the CPI score and LOA score between HIV positive and negative groups. Nearly 50% of the patients living with HIV had CPI score and LOA score greater than 2 (Tables 4 and 5).

## 4. Discussion

In this study the oral health status and prevalence of oromucosal lesion of 126 patients suffering from HIV/AIDS were compared with 532 HIV negative patients.

The study showed that nearly 85.7% of the patients suffering from HIV/AIDS belonged to lower socioeconomic status and were having less awareness about oral health maintenance. Nearly 80% were under antiretroviral drug therapy. The lack of awareness and effect of antiretroviral drugs could be responsible for depleted oral health of HIV positive individuals.

Higher mean DMFT score of HIV positive individuals indicates their poor oral health status and warrants the need of special attention towards it. A higher missing teeth (MT) component of DMFT and lower FT component indicate that the extraction was the treatment that has been carried out mostly as compared to the restorative care.

Progression of HIV infection is associated with a range of oral manifestations. Oral lesions have been widely studied and some were found to have diagnostic and prognostic values [6, 7].

Nearly 75% of the people suffering from HIV presented with some form of oromucosal condition. Candidiasis was the most common condition observed in HIV patients. Similar findings were reported by Gillespie and Marino (1993) and [8] Patton et al. (2002) [9]. They also found candidiasis as the most common oral lesion affecting HIV people.

Nearly 50% of people suffering from HIV had CPI score and LOA score >2. This indicates poor periodontal health in patients suffering from HIV. Similar to our study, Ranganathan et al. (2007) [10] found greater severity and extent of periodontal breakdown in 136 south Indian human immunodeficiency virus seropositive patients than in normal controls.

Antiretroviral therapy has changed the course of HIV disease and improved quality of life in HIV patients but the patients may also experience adverse effects [11]. The orofacial adverse effects of HAART including oral ulcers, xerostomia, mucositis, hyperpigmentation, erythema multiforme (EM), cheilitis, perioral paresthesia, angioedema, and taste alteration have been reported [12]. The prevalence of high oromucosal lesion and poor oral health including periodontal disease may be an attribute to the prolonged consumption of antiretroviral drugs.

## 5. Conclusion

The study shows that HIV positive patients have poor oral health status compared to the HIV negative individuals. Majority of the people living with HIV belong to lower socioeconomic status and also have less awareness about oral health. The use of antiretroviral drugs further depletes their oral health and is responsible for development of oromucosal lesions. Candidiasis was the most common oral lesion found in HIV positive patients. Effective policies need to be drafted to take care of the oral health of this high risk group. The need for government intervention, nongovernmental organizations, and public private partnership is required for oral health promotion of this high risk group.

## Figures and Tables

**Table 1:** Sociodemographic data, oral hygiene practices, and adverse habits of HIV positive and negative individuals.

Factor	Categories	HIV positive *N* (%)	HIV negative *N* (%)	*P* value
Age (years)^t^	Mean ± SD	36.99 ± 9.24	39.62 ± 15.47	0.068

Gender^#^	Male	76 (60.3%)	300 (56.4%)	0.484
	Female	50 (39.7%)	232 (43.6%)	

Socioeconomic status^#^	Upper class	0 (0%)	74 (13.9%)	<0.0001∗
	Middle class	18 (14.3%)	280 (52.6%)	
	Lower class	108 (85.7%)	178 (33.5%)	

Oral hygiene practices^#^	Toothbrush and paste	83 (65.9%)	450 (84.6%)	<0.0001∗
	Toothbrush and powder	19 (15.1%)	37 (7%)	
	Other methods (burnt tobacco, finger, ash, datun, etc.)	24 (19%)	45 (8.5%)	

Adverse habits^#^	Tobacco (smoking or smokeless form)	55 (43.7%)	227 (42.7%)	0.945
	Alcohol consumption	24 (19%)	98 (18.4%)	
	No deleterious habits	47 (37.3%)	207 (38.9%)	

Drug use^#^	No drug consumption	26 (20.6%)	295 (55.5%)	<0.0001∗
	<5 years	25 (19.8%)	84 (15.8%)	
	5–10 years	13 (10.3%)	61 (11.5%)	
	>10 years	62 (49.2%)	92 (17.3%)	

*P value < 0.05 is statistically significant.

^t^: independent sample *t* test.

^#^Chi-square test.

**Table 2:** Dentition status of the HIV positive and negative group.

Factor	HIV positive mean (SD)	HIV negative mean (SD)	*P* value
Decayed teeth (DT)	4.90 (4.5)	2.43 (1.54)	<0.0001∗
Missing teeth (MT)	6.30 (5.21)	3.93 (2.8)	<0.0001∗
Filled teeth (FT)	1.63 (0.64)	1.98 (1.5)	0.012∗
DMFT score	12.83 (9.6)	8.34 (7.6)	<0.0001∗

*P value < 0.05 is considered statistically significant.

**Table 3:** Oral-mucosal lesions between HIV positive and negative groups.

Condition	HIV nositive *N* (%)	HIV negative *N* (%)	*P* value
No abnormal condition	32 (25.4%)	353 (66.35%)	<0.0001∗
Malignant tumor	6 (4.76%)	0 (0%)	
Leukoplakia	12 (9.8%)	60 (11.28%)	
Lichen planus	7 (5.55%)	55 (10.34%)	
Ulceration	15 (11.90%)	12 (2.26%)	
Candidiasis	46 (36.51%)	18 (3.38%)	
Abscess	5 (3.97%)	16 (3.00%)	
Other conditions	3 (2.38%)	18 (3.38%)	

*P value < 0.05 is considered statistically significant.

**Table 4:** CPI scores (community periodontal index) between HIV positive and negative groups.

Category	CPI score 0	CPI score 1	CPI score 2	CPI score 3	CPI score 4	*P* value
HIV positive	1 (0.8%)	19 (15.1%)	39 (31%)	27 (21.4%)	40 (31.7%)	<0.0001∗
HIV negative	111 (20.9%)	183 (34.4%)	59 (11.1%)	118 (22.2%)	61 (11.5%)	

*P value < 0.05 is considered statistically significant.

**Table 5:** LOA scores (loss of attachment) between HIV positive and negative groups.

Category	LOA score 0	LOA score 1	LOA score 2	LOA score 3	LOA score 4	*P* value
HIV positive	9 (7.1%)	5 (4%)	33 (26.2%)	47 (37.3%)	32 (25.4%)	<0.0001∗
HIV negative	115 (21.6%)	226 (42.5%)	72 (13.5%)	58 (10.9%)	61 (11.5%)	

*P value < 0.05 is considered statistically significant.
